# Targeted gene therapy of nasopharyngeal cancer *in vitro *and *in vivo *by enhanced thymidine kinase expression driven by human TERT promoter and CMV enhancer

**DOI:** 10.1186/1756-9966-29-94

**Published:** 2010-07-13

**Authors:** Cong-Xiang Shen, Zhong Wen, Yu-Hong Qian, Shao-Feng Mu, Xiao-Fang Guan

**Affiliations:** 1 Otolaryngology-Head & Neck Surgery, Zhujiang Hospital, The Southern Medical University, Guangzhou 510282, China

## Abstract

**Background/Aim:**

To explore the therapeutic effects of thymidine kinase (TK) expressed by enhanced vector pGL3-basic- hTERTp-TK-EGFP-CMV driven by human telomerase reverse transcriptase promoter (hTERTp) as well as cytomegalovirus immediate early promoter enhancer (CMV).

**Materials/Methods:**

Enhanced TK-EGFP expression was confirmed by fluorescent microscopy, real time PCR and telomerase activity. Its effects were examined by survival of tumor cells NPC 5-8F and MCF-7, index of xenograft implanted in nude mice and histology.

**Results:**

Compared with non-enhanced vector pGL3-basic-TK-hTERTp-EGFP, TK expressed by the enhanced vector significantly decreased NPC 5-8F and MCF-7 cell survival rates after ganciclovir (GCV) treatment (p < 0.001) and tumor progress in nude mice with NPC xenograft and treated with GCV, without obvious toxicity to mouse liver and kidney.

**Conclusion:**

The enhanced TK expression vector driven by hTERTp with CMV enhancer has brighter clinical potentials in nasopharyngeal carcinoma therapy than the non-enhanced vector.

## Introduction

Nasopharyngeal carcinoma (NPC) is one of highly prevalent, most harmful malignant tumors in Southern China and Southeast of Asia. It is caused by the interaction between genetic background and environmental factors such as Epstein-Barr virus. At present, radiotherapy and/or induction chemotherapy is the mainstay of treatment modalities. Despite continuously progress in radiotherapeutic equipment and technology, the 5-year survival rate of NPC remains about 50% without fundamental improvement over the past several decades. Understanding the etiology and developing new effective therapeutic modality are particularly important in NPC treatment.

Suicide gene therapy is a promising modality for cancer treatment. Such therapy introduces a drug susceptible gene such as herpes simplex virus thymidine kinase (TK) gene into tumor cells. Expressed TK phosphorylates its substrate, a nontoxic prodrug ganciclovir (GCV), leading to accumulation of the toxic ganciclovir triphosphate and cell apoptosis. The ideal suicide gene expression constructs should have high specificity and killing efficacy to tumor cells. To selectively introduce suicide gene into tumor cells, many tumor specific promoters have been employed to construct tumor-specific suicide gene expression vectors.

Human telomerase reverse transcriptase (hTERT), the core component of telomerase, plays important roles in vast majority of malignant tumors including nasopharyngeal carcinoma. The telomerase activity and level of hTERT expression are enhanced in all nasopharyngeal carcinoma cell lines and 88% nasopharyngeal tissues. Their activities are closely correlated with clinical biological characteristics of nasopharyngeal carcinoma[1,2]. Therefore, telomerase/hTERT is utilized as a targeted gene for treatment of nasopharyngeal carcinoma and its promoter has been widely employed to drive the tumor-specific expression of exogenous genes. For example, Wang et al[3] and Zhang et al [4] constructed vectors pGL3-hTp-TK/GCV and TERT-E1A-TK, respectively, both of which can kill lung cancer cells and transplanted tumor *in vitro *and *in vivo*. Zheng et al [5] constructed vector pHSV-TK/CRAD, which can significantly enhance the killing effect of GCV on liver cancer in animal. Shen et al [6] selectively expressed shRNA in nasopharyngeal carcinoma cells by introducing hTERT, which successfully inhibited telomerase activity and induced cell apoptosis. We [7] have reported previously that administration of antisense oligodeoxynucleotide of telomerase RNA (hTR) and hTERT subunit can inhibit telomerase in tumor cells and induce tumor cell apoptosis. Recently, we [8,9] exploited the hTERT promoter to construct pGL3-hTERTp-TK vector and introduced the vector into NPC tumor cells *in vitro *and *in vivo *in mice xenograft, which killed NPC tumor cells and xenograft without observing toxicity to liver and kidney. However, its tumor killing efficacy is less than that of non-TK targeted system driven by cytomegalovirus immediate early promoter (CMV). To further increase TK mediated tumor killing efficacy and facilitate tracing TK expression, we constructed a new vector by inserting a CMV enhancer and an EGFP reporter gene into pGL3-hTERTp-TK vector, and evaluated its therapeutic efficacy in *in vitro *and *in vivo *tumor therapy.

## Materials and methods

### 1. Reagents

Fetal bovine serum (FBS) was purchased from Hangzhou Sijiqing Company. PCR kit and TaqMan real time PCR kit were from Takara Bio-engineering Co., Ltd. 3-(4,5-Dimethylthiazol-2-yl)-2,5-diphenyltetrazolium bromide (MTT) was from Sigma, USA; Ganciclovir (GCV) was from ROCH company. Lipofectamine 2000, DMR IE2C and Trizol were from Invitrogen. TRAPEZE^® ^RT telomerase activity detection kit was purchased from KeyGen (Nanjing, China). Plasmid Midi Kit was from Heda Biotech (Guangzhou, China). All PCR primers were synthesized by Shanghai Ying-Jun Biotechnology Co., Ltd.

### 2. Cell lines

Human nasopharyngeal carcinoma 5-8F cells (NPC 5-8F), human breast cancer cells MCF-7 and human vascular endothelial cells ECV were kindly provided by Department of Cell Biology, the Southern Medical University, and maintained in RPMI 1640 supplemented with 10% heat-inactivated fetal bovine serum at 37°C in a 5% CO_2 _incubator (Shell LAB, USA) as previously reported [10].

### 3. Construction of plasmid with luciferase reporter gene

EGFP gene was obtained from pEGFP-N1 by PCR using forward primer Egfp-F: CCCAAGCTTATGGTGAGCAAGGGCGAGGAG and reverse primer Egfp-R: GCTCTAGATTACTTGTACAGCTCGTCCATGC. 406 bp CMV enhancer fragment was obtained from pEGFP-N1 by PCR using forward primer hCMVen-F: 5-CGGGATCCCGCGTTACATAACTTACGGT-3' and reverse primer hCMVen-R: 5-ACGCGTCGACCAAAACAAACTCCCATTGAC-3. 1131 bp TK gene with NCBI accession number AY575228 was obtained from pMD18-TK by PCR using forward primer 5-CCGCTCGAGATGGCTTCGTACCCCTGC-3' and reverse primer 5-CCCAAGCTTGTTAGCCTCCCCCATCTC-3. The 260 bp hTERT promoter was obtained from pMD18-T-hTERTp using forward primer hTERTp-F: 5-GGGGTACCAGTGGATTCGCGGGCACAGACG-3' and reverse primer hTERTp-R: 5-CCGCTCGAGAGGGCTTCCCACGTGCGCAGCA-3. All PCR fragments were verified by DNA sequence analysis. Stop codon TGA of TK gene was removed in TK reverse primer to facilitate the construction of TK-EGFP fusion protein. EGFP fragment was digested with Hind III and Xba I and subcloned into pGL3-basic plasmid to obtain pGL3-basic-EGFP. TK fragment was excised with Hind III and Xho I and subcloned into pGL3-basic-EGFP to construct pGL3-basic- TK-EGFP. hTERTp fragment was subcloned into pGL3-basic-TK-EGFP at Kpn I and Xho I sites to construct pGL3-basic-TK-hTERTp-EGFP. CMV enhancer fragment was inserted into pGL3-basic-TK-hTERTp-EGFP at BamH I and Sal I site according to previous reports [11,12] to construct the enhanced vector pGL3-basic-hTERTp-TK- EGFP-CMV. All plasmids were verified by restriction enzyme digestion.

### 4. Cell transfection as well as expression of TK-EGFP fusion protein

1 × 10^6 ^NPC 5-8F, MCF-7 and ECV cells at logarithmic phase were inoculated into one well of 6-well plate with duplications, respectively. 12 hours after inoculation, cells at about 80% confluency were transfected with 4 μg of plasmid pGL3-basic-hTERTp-TK-EGFP-CMV or pGL3-basic-hTERTp-TK-EGFP by mixed with 4 μl Lipofectamine 2000 according to the protocol provided by the manufacturer. 24 hours after transfection, the expression of TK-EGFP fusion protein was directly observed with fluorescent microscopy (Nikon Eclipsete 2000-U, USA).

### 5. RNA Isolation and TK mRNA level detection by quantitative real-time PCR

48 hours after transfection, total RNA was extracted with Trizol (Invitrogen) following the manufacturer's instruction. 4 μL mRNA of each sample was used as template in quantitative real-time PCR performed in an ABI 7500 Real-Time PCR system using Taqman PCR kit based on the manufacturer's protocol. The specific primers used in these reactions were followings: TK forward 5'-AGCAAGAAGCCACGGAAGTC-3' and reverse 5'-AGTTGCGTGGTGGTGGTTTT-3'; human β-actin forward 5'-GCATGGGTCAGAAGGATTCCT-3' and reverse 5'-TCGTCCCAGTTGGTGACGAT-3'. Relative levels of TK gene expression were normalized to β-actin mRNA level.

### 6. Telomerase activity measurement

NPC 5-8F cells at logarithmic phase were inoculated into three wells of a 6-well plate with 1 × 10^6^/well. Twelve hour later, two wells of cells were transfected with 8 μg pGL3-basic- hTERTp-TK-EGFP-CMV plasmid. Twelve hours after transfection, one well of cells transfected with pGL3-basic-hTERTp-TK-EGFP-CMV were treated with 10 μg/mL GCV. 48 hours after drug treatment, telomerase activities of all three well of cells were measured using PCR-based TRAP telomerase activity detection kit. As control, telomerase activity of 1 × 10^6 ^ECV cells at logarithmic phase was also detected using the same method. The PCR products were separated on 12% non-PAGE and visualized by silver stain.

### 7. Cell survival rate measurement by MTT method

NPC 5-8F cells at logarithmic phase were inoculated into 15 wells of 96-well plate with 1 × 10^5 ^cells in each well. Twelve hours later, 3 wells of NPC 5-8F cells were used as blank, 3 wells were transfected with 2.4 μg pGL3-basic-EGFP as control, 6 wells were transfected with pGL3-basic- hTERTp-TK-EGFP-CMV. Twelve hours after transfection, control group and three wells of the cells transfected with pGL3-basic-hTERTp-TK-EGFP-CMV were treated with 10 μg/mL GCV. 72 hours after treatment, all cells were subjected to MTT assay as described previously [10]. In detail, 20 μl of 5 g/L MTT solution was added into each well of the 96-well plate, and the plate was incubated for 4 hours at room temperature. After the culture solution was removed, 150 μl DMSO was added into each well and oscillated for 10 minutes. Then the absorption at 570 nm was measured with Startfax 2100 microplate reader (USA). Cell survival rate was expressed as A/B×100%, where A was the absorption from cells transfected with plasmid and treated with or without GCV, and B was that from the blank group.

The same experiment was performed using MCF-7 cells instead of NPC 5-8F cells.

### 8. In vivo animal experiments

Healthy male and female nude BALB/c nu/nu mice of age 4-5 weeks, weighing between 18-22 g, were from the Experimental Animal Centre of The Southern Medical University, and maintained in a SPF level aseptic environment. The animals were free access to aseptic rodent diet and water. The protocol of animal experiments was approved by ethical and humane committee of Zhujiang Hospital, The Southern Medical University.

NPC 5-8F cells at logarithmic phase were prepared as 5 × 10^6 ^cells/mL single cell suspension in phosphate buffered saline (PBS) and 0.2 ml of cell suspensions were subcutaneously inoculated into the left flank of BALB/c nude mice. The cancer growth was monitored every 3 days starting at the day after inoculation by calipers to record the length (a) and width (b), and tumor volume were calculated by the formula V = 1/2 (a × b^2^). When majority tumors reached 1.2 ~ 1.5 cm in diameter at day 10 after inoculation, nude mice were randomly divided into 6 groups: blank group, Lipofectamine group, non-enhanced group, enhanced group, enhanced/GCV group, and GCV group. Mice in blank and GCV groups were intratumorally injected with PBS; mice in Lipofectamine group were intratumorally injected 25 μL Lipofectamine alone; mice in non-enhanced group were intratumorally injected with mixture of 25 μL Lipofectamine with 10 μg plasmid pGL3-basic-hTERTp-TK-EGFP; mice in enhanced and enhanced/GCV groups were injected with the mixture of 25 μL Lipofectmine 2000 and 10 μg plasmid pGL3-basic-hTERTp-TK-EGFP-CMV. All injections were performed repeatedly at the days 4, 7, 10 and 14 after the first injection. Meanwhile, mice in GCV and enhanced/GCV groups were intraperitoneally injected 100 mg/kg bodyweight GCV every 2 days starting at day 1 after the first injection of the mixture for total 12 times. When the tumor volume reached 6 cm^3 ^in mice from blank group, all mice were sacrificed by cervical dislocation and the whole tumors were removed and weighed, and livers and kidneys from mice in Lipofectamine, enhanced/GCV and GCV groups were preserved for further histopathological examination. The inhibition rate of different treatment on tumor growth was calculated according to the following formula:

### 9. Histopathological examination

The preserved livers and kidneys were fixed with 10% formaldehyde solution and the sections were stained with hematoxylin and eosin, and analyzed by light microscopy.

### 10. Statistical analysis

Data were analyzed with SPSS11.0 statistical software and expressed as mean ± standard deviation. Statistical significant was analyzed using one-way ANOVA and q test. A p value less than 0.05 was considered as statistical significance.

## Results

### 1. Identification of pGL3-basic- hTERTp -TK-EGFP-CMV plasmid

We constructed an enhanced TK expression vector as described in the Materials and Methods section. First we examined whether we successfully constructed the enhanced TK expression vector. Digestion with BamH I and Sal I, Xho I and Xba I, Kpn I and Hind III resulted in 406 bp, 1850 bp and 1400 bp fragments, respectively, as expected. The sequences of TK gene, hTERTp and CMV enhancer have been confirmed by direct DNA sequences.

### 2. Fluorescent level of TK-EGFP gene expression

**Figure 1:**
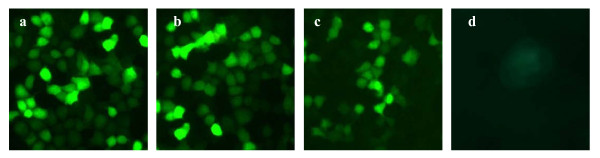
**TK gene expression detected with fluorescent microscopy**. Shown here are the cells 24 hours after transfection under fluorescent microscope (×100). (a) NPC 5-8F cells transfected with pGL3-basic-hTERTp-TK-EGFP-CMV; (b) MCF-7 cells transfected with pGL3-basic-hTERTp-TK-EGFP-CMV; (c) NPC 5-8F cells transfected with pGL3-basic-TRETp-TK-EGFP; (d) ECV cells transfected with pGL3-basic-hTERTp-TK- EGFP.

Then we measured the fluorescent level of TK-EGFP gene expression in NPC 5-8F and MCF-7 cells transfected with either the enhanced plasmid pGL3-basic-hTERTp-TK-EGFP-CMV or the non-enhanced pGL3-basic-hTERTp-TK-EGFP by observing the fluorescent intensity of co-expressed GFP under fluorescent microscope. As shown in Figure 1, NPC 5-8F and MCF-7 cells transfected with the enhanced plasmid showed very strong green fluorescence (Figure 1a and 1b). NPC 5-8F cells transfected with the non-enhanced plasmid also had very strong green fluorescence (Figure 1c). However, compared with cells transfected with the enhanced plasmid, the fluorescent intensity was decreased. ECV cells transfected with the enhanced plasmid only showed weak, flurry fluorescence (Figure1d) under the same condition. Since the expression of TK-EGFP was controlled by hTERT promoter, therefore it was only expressed in telomerase-positive cells. Furthermore, TK was fused to EGFP, expression level of EGFP not only reflected the transfection efficient, but also indirectly indicated the relative expression level of TK.

### 3. Enhanced TK mRNA level in cells transfected with pGL3-basic-hTERTp-TK- EGFP-CMV

**Figure 2:**
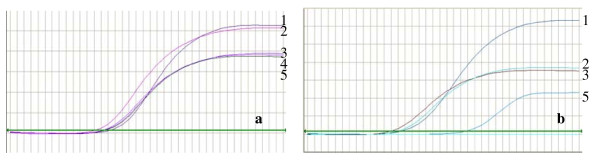
**Amplification curves of fluorescence quantitative PCR**. Shown are the amplification curves of β-actin (a) and TK gene (b) from NPC 5-8F cells transfected with pGL3-basic-hTERTp-TK-EGFP-CMV plasmid (1), from MCF-7 transfected with pGL3-basic-hTERTp-TK-EGFP-CMV plasmid (2), from NPC 5-8F cells transfected with pGL3-basic-hTERTp-TK-EGFP plasmid (3), from NPC 5-8F cells transfected with pGL3-basic-TK-EGFP plasmid (4), and from ECV cells transfected with pGL3-basic-hTERTp-TK-EGFP-CMV plasmid (5).

We further quantitatively examined the expression of TK gene in NPC 5-8F and MCF-7 cells at mRNA level by real-time PCR. Figure 2 showed the amplification curves of housekeeping gene (β-actin and TK gene, and Table 1 showed the relative expression level of TK gene to (β-actin gene. TK gene expression in NPC 5-8F, MCF-7 and ECV cells transfected with the enhanced plasmid was 4.2-fold, 2.5-fold, and 0.0027-fold of β-actin, respectively. By contrast, the TK expression level in NPC 5-8F cells transfected with pGL3-basic-hTERTp-TK-EGFP was only 0.82-fold of β-actin. No TK expression was detected in NPC 5-8F cells transfected with pGL3-basic-EGFP as expected. These results are consistent with that of Figure 1.

**Table 1:** Expression of TK gene detected with real-time PCR

Sample	Copy number (β-actin)	Copy number (TK)	Relative folds to β-actin
1	6.67E+07	2.78E+08	4.16792*
2	4.50E+07	1.13E+08	2.51111**
3	7.76E+07	2.17E+05	0.00279639
4	8.21E+07	Undetermined	Undetermined
5	1.69E+08	1.39E+08	0.822485

**Figure 3:**
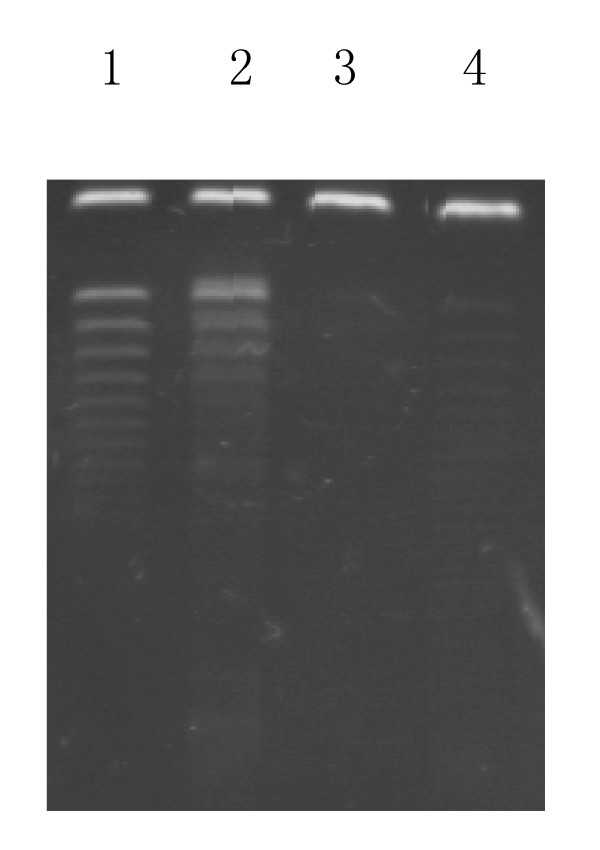
**GCV treatment down-regulates telomerase activity in 5-8F cells transfected with pGL3-basic-hTERTp-TK-EGFP-CMV**. Shown are the silver stain visualized PCR products of telomerase activities assay by PCR-based TRAP telomerase activity detection kit from NPC 5-8F cells transfected with enhanced plasmid pGL3-basic-hTERTp-TK-EGFP-CMV (lane 1), NPC 5-8F cells without transfection (lane 2), 5-8F cells transfected with pGL3-basic-hTERTp-TK-EGFP-CMV and treated with GCV (lane 3), and ECV cells itransfected with pGL3-basic-hTERTp-TK-EGFP-CMV and treated with GCV (lane 4).

Numbers 1, 2, 3, 4, 5 correspond to the numbers in Figure 3.

1: NPC 5-8F cells transfected with pGL3-basic- hTERTp-TK- EGFP- CMV;

2: MCF-7 transfected with pGL3-basic- hTERTp-TK- EGFP- CMV;

3: NPC 5-8F cells transfected with pGL3-basic- hTERTp-TK- EGFP;

4: NPC 5-8F cells transfected with pGL3-basic -TK-EGFP;

5: ECV cells transfected with pGL3-basic- hTERTp-TK- EGFP- CMV.

Data are presented as mean ± standard deviation from these experiments. *P < 0.0001 for sample 1 vs sample 3, sample 1 vs sample 5 and sample 2 vs sample 3. **P < 0.001 for sample 2 vs sample 5.

### 4. Reduced telomerase activity by pGL3-basic-hTERTp-TK- EGFP-CMV/GCV

Next we examined telomerase activity in PNC 5-8F cells transfected with the enhanced plasmid with or without GCV treatment. NPC 5-8F cells transfected with the enhanced plasmid were telomerase activity positive. However, the telomerase activity was decreased by 48 hours of GCV treatment. As control, ECV cells showed weak telomerase positive (Figure 3).

### 5. Decreased survival rate of tumor cells transfected with the enhanced plasmid and treated with GCV

Having confirmed that transfection of the enhanced plasmid increased the expression of TK, we further studied whether transfection of the enhanced plasmid could affect the effect of GCV on the survival rate of nasopharyngeal carcinoma NPC 5-8F cells and breast cancer MCF-7 cells by using MTT method. As shown in Tables 2 and 3, compared with non-transfected, untreated cells, transfection of control plasmid pGL3-basic-EGFP had no effect on survival rates of tumor cells 5-8F and MCF-7 with GCV treatment, and transfection of the enhanced plasmid pGL3-basic- hTERTp-TK-EGFP-CMV alone did not change the survival rates of tumor cells NPC 5-8F and MCF-7. However, after GCV treatment, survival rates of NPC 5-8F and MCF-7 cells transfected with the enhanced plasmid decreased to 0.370 ± 0.024 and 0.462 ± 0.049, respectively, which was significantly lower than that, 0.533 ± 0.020 and 0.515 ± 0.025, of tumor cells NPC 5-8F and MCF-7 transfected with the plasmid pGL3-basic-hTERTp-TK- EGFP and treated with GCV, respectively.

**Table 2:** PNPC cell survival rates measured by MTT assay

Codes and Samples	Survival rates
A. Cells without treatment	1
B. Cells transfected with pGL3-basic-EGFP and with GCV treatment	0.984 ± 0.009
C. Cells transfected with pGL3-basic- hTERTp-TK-EGFP-CMV and treated with GCV	0.370 ± 0.024*
D. Cells transfected with pGL3-basic-hTERTp-TK-EGFP-CMV without GCV	0.982 ± 0.010
**E. Cells transfected with pGL3-basic-hTERTp-TK-EGFP and treated with GCV**	0.533 ± 0.020*

Data are expressed as mean ± standard deviation from three experiments. * indicates p < 0.0001 compared with other groups

**Table 3:** MCF-7 cell survival rates measured by MTT assay

Codes and Samples	Survival rates
A. Cells without treatment	1
B. Cells transfected with pGL3-basic-EGFP and with GCV treatment	0.987 ± 0.006
C, Cells transfected with pGL3-basic-hTERTp-TK-EGFP-CMV and treated with GCV	0.462 ± 0.049*
D. Cells transfected with pGL3-basic-hTERTp-TK-EGFP-CMV without GCV	0.984 ± 0.011
**E. Cells transfected with pGL3-basic-hTERTp-TK-EGFP and treated with GCV**	0.515 ± 0.025*

Data are expressed as mean ± standard deviation from three experiments. * indicates p < 0.0001 compared with other groups

### 6. Injection of pGL3-basic-hTERTp-TK-EGFP-CMV/GCV inhibited tumor progress in vivo

**Figure 4:**
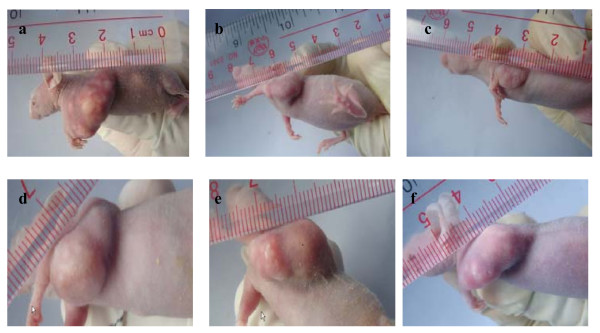
**Tumor inhibition of pGL3-basic-hTERTp-TK-EGFP-CMV/GCV in nude mice with NPC xenograft**. Shown are the NPC xenograft in nude mice without treatment (a), injected with GCV and the non-enhanced plasmid (b), injected with GCV and the enhance plasmid (c), injected with GCV(d), injected with Lipofectamine 2000 (e) and injected with the enhance plasmid without GCV (f).

Then we explored whether injection of pGL3-basic-hTERTp-TK-EGFP -CMV/GCV could inhibit tumor progress. As showed in Figure 4 and table4, nude mice inoculated NPC 5-8F cells developed tumor with volume of 6.23 ± 0.04 cm^3 ^and weight of 2.68 ± 0.02 g. After injection of non-enhanced plasmid and GCV, the tumor volume and weight decreased to 3.51 ± 0.02 cm^3 ^and 1.51 ± 0.01 g (p = 0.000), respectively. In comparison, after injection of the enhanced plasmid and GCV, the tumor volume and weight decreased to 2.27 ± 0.02 cm^3 ^and 1.17 ± 0.01 g, respectively, which were significantly lower than those of nude mice injected with the non-enhanced vector (p = 0.000). The inhibition rates of tumor progress were 43.68% and 56.34% for injection of non-enhanced and enhanced plasmids, respectively.

**Table 4:** Injection of pGL3-basic- hTERTp-TK- EGFP- CMV/GCV inhibited tumor development in vivo

Sample	Animals	Tumor volume at day 39 (cm^3^)	Tumor weight at day 39 (g)	Inhibition rate
Blank	5	6.23 ± 0.04	2.68 ± 0.02	/
Non-enhanced group	5	3.51 ± 0.02	1.51 ± 0.01	43.68%*
Enhanced group/GCV	5	2.72 ± 0.02	1.17 ± 0.01	56.34%*

Enhanced group	5	5.80 ± 0.13	2.51 ± 0.05	6.48%*

GCV group	5	5.98 ± 0.09	2.56 ± 0.09	4.32%*

**Lipofectamine group**	5	5.83 ± 0.14	2.51 ± 0.02	6.41%*

Data are expressed as mean ± standard deviation from three experiments. * indicates p < 0.0001 compared with the other group.

### 7. Injection of pGL3-basic-hTERTp-TK-EGFP-CMV/GCV had no toxicity to liver and kidney of nude mice

We further examined whether injection of GCV and the enhanced plasmid could have any toxicity to nude mouse. No obvious damages were observed in H&E stain in the livers and kidneys from nude mice in GCV/enhanced, GCV and Lipofectamine 2000 groups.

## Discussions

Molecularly targeted therapy is a promising research area in cancer therapy. Application of suicide gene in tumor therapy was limited due to lack of selectivity. Suicide gene TK or CD expression system driven by tumor-specific promoter has overcome the disadvantage and become a powerful modality in cancer therapy.

Identification of molecular targets is the key in molecularly targeted therapy. Molecules involved in carcinogenesis, cancer gene mutation, tumor angiogenesis and tumor signal transduction, telomerase, and growth factors such as epidermal growth factor are potential targets for tumor treatment. Gene mutation [13], EB virus [14], telomerase [1] and nasopharyngeal cancer stem cells [10,15,16] are reportedly involved in the progress of nasopharyngeal cancer. Therapies targeted to the molecules and molecules related to those mentioned above have made primary progress in nasopharyngeal cancer treatment [14,8,10]. We have found that introduction of TK expression vector driven by hTERT promoter (hTERTp/TK) could kill nasopharyngeal carcinoma cells, nasopharyngeal carcinoma stem cells, and nasopharyngeal tumor xenograft in nude mice without side effects on cultured normal cells and damaging mouse liver and kidney functions [17]. Studies on other tumors also confirmed the efficacy of hTERTp/TK for cancer therapy. Introduction of herpes simplex TK gene expression virus vector driven by hTERT promoter (AdhTERT/TK) can specifically kill the undifferentiated thyroid tumor and thyroid tumor xenograft in nude mice, enhance the tumor GCV sensitivity without toxic reaction in liver and the whole body examined by liver pathology and serum enzymology [18]. By contrast, introduction of TK gene expression vector driven by CMV promoter (CMV/TK) not only kills tumor xenograft, but also demonstrates obvious liver pathological changes and damaged liver function revealed by serum enzymology. In addition, hTERT promoter has been used to target other tumor killing factors, such as caspase 8, TRAIL and Bax, and subsequently induces tumor specific apoptosis [19,18-23] and enhances the sensitivity of tumor cells to GCV without adverse effect. Thus, targeted gene therapy remains a highly promising system and progress in this field is gaining momentum.

An ideal targeted vector should have both good tumor specificity and high killing efficacy. To improve the killing efficacy, double suicide gene, dual-promoter, a promoter plus an enhancer and double enhancers, etc. have been employed. Kong et al [24] have exploited adenovirus-mediated TK/CD double suicide genes, which are more effective in killing breast cancer cells *in vitro*. Huang et al [25] have excised TK/CD suicide gene therapy with combination of radiotherapy to enhance radio-sensitivity of tumor. Liao et al [26] have found that radiation can enhance therapeutic efficacy of hTERTp-mediated gene therapy. But hTERT/CMV dual promoter vector can not increase the activity of the promoter due to possible interference between two promoters resulting in the decreased efficacy. CMV enhancer has been widely used to improve the suicide gene expression driven by hTERT promoter and has application potentials in targeted cancer gene therapy. Wang [11] has explored the effects of hTERTp/CMV-regulated TK/CD system in five tumor cell lines and found that adding CMV enhancer increases TK/CD expression level by 3~26 times without affecting hTERTp-mediated targeting. Further study in HeLa cells [12] has revealed that enhancers can improve hTERT promoter activity by 6~13 times, among which SV40-CMV dual enhancer/hTERT promoter has the highest activity, which is nearly 3-fold of CMV enhancer/hTERT promoter; two hTERTp regulated CD/TK fusion suicide gene driven by SV40/CMV dual enhancer has very high specificity and efficacy to tumor cells. Other vectors have also been used in cancer gene therapy. Song [27] has applied SB system, in which TK gene expression is targeted to cancer cells by hTERTp and enhanced by SV40 enhancer, to selectively kill liver cancer cells. In the present paper, TK was fused to EGFP for conventional observation of transfection efficiency. In addition, because hTERT only expressed in telomerase positive cells, stronger fluorescent signal reflect the relative expression of TK protein, indicating that we have successfully constructed a CMV enhanced-, hTERTp driven-TK gene expression vector, pGL3-basic-hTERTp-TK-EGFP-CMV and increased the activity of hTERT promoter and expression of its downstream TK gene.

Our results indicate that transfection of the enhanced pGL3-basic-hTERTp-TK-EGFP-CMV with GCV treatment significantly inhibits the survival rate of nasopharyngeal carcinoma 5-8F cells and the progress of nasopharyngeal xenograft in nude mice, and the enhanced pGL3-basic-hTERTp-TK-EGFP-CMV/GCV has much better tumor killing efficacy than pGL3-basic-hTERTp-TK-EGFP/GCV in both NPC 5-8F and MCF-7 cells. Quantitative fluorescence PCR showed that TK expression level was increased by 2 to 5-fold in NPC 5-8F and MCF-7 cells transfected with the enhanced vector compared with that in the cells transfected with non-enhanced vector. By contrast, TK expression was not altered by transfection of the enhanced vector in telomerase negative ECV cells. No side effects on liver and kidney by pathological examination were observed in *in vivo *experiment, suggesting the effects of the enhanced vector is specific to tumor cells and it is safe in *in vivo *application.

The mechanism by which hTERTp/CMV-dual-regulated TK expression can enhance the targeted killing of nasopharyngeal carcinoma cells need to be further investigated. In our previous study on hTERT-TK expression vector, the killing effect of TK under hTERT promoter, which is a much weaker than CMV promoter, is significantly reduced compared with that of TK under the non-selective promoter CMV. In consistence with our other reports [7-9], our results suggest that addition of CMV promoter can significantly enhance TK efficacy without changing its targeting controlled by hTERT. Wang [11,12] proposed that CMV can recognize specific binding sites of different activators, enhancers and promoters, therefore synergistically and dramatically promotes protein expression. In addition, co-effect of SV40 and CMV enhancers also enhance promoter activity because SV40 enhancer can effectively increase the amount of exogenous DNA in the nucleus. Therefore, the interference between hTERTp and CMV hindered the efficiency of vector.

In this study, we found that telomerase activities are significantly reduced in both NPC 5-8F and MCF-7 cells transfected with the enhanced vector after GCV treatment, but not changed in ECV cells transfected with the enhanced vector (Figure 4). One possible explanation is that the reduced telomerase activity in cells transfected with the enhanced vector is the result of the cell death induced by TK/GCV. We speculate that in the early stage of transfection of the enhanced vector, when GCV was not added into the cells, telomerase activity is temporally increased; after adding GCV into the cells, cell numbers dramatically decreased resulting in the reduced telomerase activity. However, we can not exclude other possibilities. Decreased telomerase activity has been shown to inhibit tumor proliferation. Transfection of eukaryotic vector containing antisense of hTERT in human gastric cancer SGC-7901 cells attenuated telomerase activity, reduced telomere length, decreased expressions of hTERT, bcL-2 and c-myC at mRNA and protein levels without changing hTR and TP1 expression, inhibited cell proliferation and arrested the cells in G0/G1 phase [28]. Injection of SGC-7901 cells transfected with the eukaryotic vector containing antisense of hTERT did not induce tumor development in nude mice, whereas injection of control cells without transfection induced touchable tumor growth. Transfection of hTERT small interfering RNA had similar results [29]. But it is more plausible that the mechanisms by which hTERT antisense or siRNA induced tumor apoptosis through reduced telomerase activity are different from that of the direct tumor killing of TK gene expression driven by hTERT promoter.

To our knowledge, the effect of TK gene expression driven by CMV enhancer/hTERT promoter has not been previously studied in NPC. In the present study, we also improved the methodology: 1) We applied co-expression of TK gene with EGFP in favor of the experimental observation, and adopted real time fluorescence quantitative PCR to measure the difference in TK gene expression; 2) We studied tumor-inhibitory effect of the enhanced vector both *in vitro *and *in vivo*, and explored the safety of *in vivo *application of the enhanced vector; 3) The enhanced vector had similar tumor inhibitory efficacy in both NPC and MCF-7 cells, suggesting it might have broader application potentials in other cancer therapies; 4) We compared the effect of the enhanced vector with the single promoter vector to elucidate the increased efficacy of the enhanced vector/GCV system. As an enhanced targeting vector, transfection of pGL3-basic-hTERTp-TK-EGFP-CMV has obvious targeted killing efficacy on nasopharyngeal carcinoma and breast cancer, but its application in other tumor therapies need to be further investigated.

In conclusion, we successfully constructed the enhanced TK gene expression vector driven by hTERT promoter and CMV enhancer, and revealed that the enhanced vector indeed increased the TK expression and improved its killing efficacy on NPC *in vitro *and *in vivo*, indicating that the enhanced vector has clinical potentials in nasopharyngeal carcinoma gene therapy.

## Competing interests

The authors declare that they have no competing interests.

## Authors' contributions

CXS carried out the subtotal molecular genetic studies, participated in the design of the study, and performed the statistical analysis. ZW conceived of the study, and participated in its design and coordination. and drafted the manuscript. YHQ carried out the cell culture. SFM participated in the PCR, MTT, telomerase activity and DNA sequence. SFG participated in study work in vivo. All authors read and approved the final manuscript.
